# Stimulated left DLPFC-nucleus accumbens functional connectivity predicts the anti-depression and anti-anxiety effects of rTMS for depression

**DOI:** 10.1038/s41398-017-0005-6

**Published:** 2018-03-09

**Authors:** Lian Du, Huan Liu, Wanyi Du, Fenglei Chao, Lei Zhang, Kejian Wang, Chunxia Huang, Yuan Gao, Yong Tang

**Affiliations:** 1 Department of Psychiatry, The First Affiliated Hospital of Chongqing Medical University, Chongqing, 400016 P.R. China; 2 Laboratory of Stem Cells and Tissue Engineering, Chongqing Medical University, Chongqing, 400016 P.R. China; 3 Department of Histology and Embryology, Chongqing Medical University, Chongqing, 400016 P.R. China; 4 Department of Anatomy, Chongqing Medical University, Chongqing, 400016 P.R. China; 5 Department of Physiology, Chongqing Medical University, Chongqing, 400016 P.R. China; 6 Department of Geriatrics, The First Affiliated Hospital of Chongqing Medical University, Chongqing, 400016 P.R. China

## Abstract

Not all depression patients effectively respond to repeated transcranial magnetic stimulation (rTMS). We tested whether the intrinsic functional connectivity (FC) strength between the stimulated left dorsolateral prefrontal cortex (DLPFC) and left nucleus accumbens (NAcc) might predict effects of rTMS. Twenty-two medication-naïve depression patients received rTMS on left DLPFC for 2 weeks and underwent baseline functional magnetic resonance imaging (fMRI). We compared the amplitude of the low-frequency fluctuation (ALFF) and regional homogeneity (ReHo) in the stimulated target (the cortex region directly stimulated by rTMS) located in the left DLPFC, and the left NAcc, as well as the intrinsic FC of the DLPFC–NAcc between early improvers and non-improvers. We evaluated the association between the baseline brain imaging features (ALFF, ReHo, and FC) and improvements in depression and anxiety symptoms. We found that the pretreatment ALFF and ReHo in the stimulated DLPFC and left NAcc did not significantly differ between the subgroups. The early improvers displayed increased negative FC strength between the stimulated DLPFC and left NAcc with respect to non-improvers. The stimulated DLPFC–NAcc FC strength negatively correlated with improved depressive and anxious symptoms. This study is the first to demonstrate that the resting-state FC of the stimulated DLPFC–NAcc, rather than regional brain activity or local synchronization in the stimulated target, might predict the anti-depression and anti-anxiety effects of rTMS for depression.

## Introduction

Major depressive disorder (MDD) is a highly prevalent psychiatric disorder characterized by affective and cognitive symptoms. Unfortunately, approximately one-third of MDD patients do not respond to the various pharmacological and psychotherapeutic treatments available^1^. Repetitive transcranial magnetic stimulation (rTMS), which was approved by the US Food and Drug Administration in 2008, has been proposed to be one of safe and available treatments for refractory MDD^2–4^. However, not all MDD patients exhibit sufficient effective responsiveness and remission during the acute or long-term phases^5^. It is important to recognize the pretreatment characteristics of those patients who effectively respond to rTMS to determine which individuals with MDD would receive the most benefit.

The position of the rTMS-stimulated target on the skull has been considered a possible factor that influences its anti-depression effects^6^. The left dorsolateral prefrontal cortex (DLPFC)^2,7^ is one of the most popular rTMS-stimulated targets for treating MDD, which anatomically corresponds to Brodmann areas (BA) 9 and 46. It remains unclear as to which part of the left DLFPC is the optimized target for rTMS. Furthermore, the anti-depression effects of rTMS on the left DLPFC is still mild, regardless of whether the ‘5 cm’ method (with the left DLPFC target as a point located 5 cm in front of the ‘hand motor hotspot’ in the parasaggital plane pointing anteriorwards), the EEG F3 technique or the neuronavigation technique are used^8–10^. Therefore, it is necessary to find a new strategy to optimize the stimulated position of rTMS to promote its clinical effectiveness.

The effects of rTMS have been proved to propagate beyond the stimulated site on the skull and even influence deep brain structures, impacting multiple neuronal circuits in MDD^11–13^. Recently, Fox and colleagues analyzed published data acquired from normal subjects to reveal that the previously reported anti-depression efficacy of rTMS was related to the strength of resting-state functional connectivity (FC), which was defined as temporal correlations of functional magnetic resonance imaging (fMRI) signals, between the left DLPFC and the subgenual anterior cingulate cortex (sgACC), which is a target of deep brain stimulation (DBS) in cases of MDD^14,15^. This evidence suggests that both invasive (DBS) and noninvasive (rTMS) brain stimulation protocols may work effectively by activating common circuits^16^. Therefore, we further speculate that rTMS can exert its effects by affecting DBS targets if FC exists between superficial and deeper targets. The relationships between invasive and noninvasive targets can feasibly be detected by measuring intrinsic FC via resting-state fMRI. However, Fox et al. computed the average coordinates of the target for each method, which might not be completely equivalent to the actual position of an individual target. Using that approach, those authors revealed that the strength of FC between various targets in the left DLPFC and sgACC were quite different^17^. Few studies have reported whether pretreatment individual FC between the stimulated target in the left DLPFC and deep brain regions (e.g., DBS targets) can predict the anti-depression effects of rTMS.

The nucleus accumbens (NAcc), which plays a central role in the reward system, is another effective target of DBS (invasive) treatment for depression^14,18^. The function of this region is related to anhedonia, which is a core symptom of melancholic MDD^19^. The NAcc also modulates activity in many other regions involved in emotion, and DBS targeting the NAcc exerts both antidepressant and antianxiety effects^20–22^. Furthermore, previous studies demonstrated that rTMS (noninvasive) over left DLPFC also had anti-depression and anti-anxiety effects in MDD^2,23,24^. It is important to explore the characteristics of the directly stimulated brain region deciding the final effect of rTMS: the regional brain activity, the local synchronization, or the FC with remote regions pretreatment? Regional brain activity is measured by blood oxygen level-dependent (BOLD) signal variations over time according to the amplitude of low-frequency fluctuation (ALFF)^25^. The ALFF encodes physiologically meaningful indicators of intrinsic brain activity, which is thought to reflect the intensity of spontaneous brain activity at rest^25^. Local synchronization, reflected by the functional coherence or synchronization of BOLD fluctuations in a given voxel with its nearest voxels, is known as the regional homogeneity (ReHo)^26^. The ReHo is neurobiologically relevant and dependent upon a combination of anatomical, developmental, and neurocognitive factors^27^. Both the ALFF and ReHo are reliable and sensitive neuromarkers in MDD^28^.

Thus, we hypothesized that greater FC strength between the stimulated left DLPFC and NAcc might predict better anti-depression and anti-anxiety effects of rTMS. In the current study, we examined the association of the baseline FC strength between the stimulated left DLPFC and left NAcc and changes in depression and anxiety symptoms after rTMS. We also compared the regional brain activity and local synchronization in the stimulated left DLPFC and left NAcc and the FC strength of the stimulated left DLPFC–NAcc between early improvers and non-improvers.

## Methods

### Participants

Twenty-two medication-free outpatients (medication-free in the previous month and medicated less than a week in total before) with a single episode of or recurrent depression were recruited for this study. The diagnosis of MDD was confirmed by the Structured Clinical Interview for the Diagnostic and Statistical Manual of Mental Disorders (SCID-I/P, Chinese version). Patients were excluded if they had a history of alcohol or drug abuse, current or past psychotic disorders, any current clinically significant neurological disorder or other serious physical diseases, morphological anomalies in the brain, and any electronic or metal implants. A consort flowchart showing the excluded participants and reasons is provided in Supplementary Fig. S1. The choice of rTMS treatment for MDD patients was decided by their clinicians, and then they were screened by researchers to assess if they meet the above inclusion/exclusion criteria. Written informed consent was obtained from all subjects according to the study protocol. The study protocol was reviewed and approved by the Local Medical Ethics Committee of the First Affiliated Hospital of Chongqing Medical University.

### rTMS procedure

rTMS was delivered in sessions by a YRD CCY-I magnetic simulator (YIRUIDE Inc., Wuhan, China). Patients received a total of ten sessions of rTMS (five sessions per week for 2 weeks). The stimulation parameters were guided by recently published international safety guidelines^29^: 100% magnetic field strength relative to the patient’s observed resting motor threshold, at ten pulses per second for 3 s, with an intertrain interval of 21 s. Treatment sessions lasted for 20 min (50 trains) and consisted of 1,500 pulses. The stimulated position in the left DLPFC was located using the ‘5 cm’ method^2,4^. Every patient was required to wear an elastic hat with specific coordinates during treatment to ensure the stimulated target was in the same position during each session.

### Clinical assessment

Patients were assessed using both the 17-item Hamilton Depression Scale (HAMD) and the 14-item Hamilton Anxiety Scale (HAMA) at baseline and at the end of the 2 weeks of treatment. The depressive/anxiety symptom improvement ratio was defined as the pre-TMS score minus the post-TMS HAMD/HAMA score divided by the pre-TMS score. We grouped the patients into early improvers (HAMD-17 scores reduced by at least 20% from baseline to endpoint) and early non-improvers (HAMD-17 scores reduced by less than 20%)^30^.

### Image acquisition

Data were acquired on a 3.0 Tesla MRI system (GE Medical Systems, Waukesha, WI, USA) at the First Affiliated Hospital of Chongqing Medical University. Subjects were scanned with the same hat that was used during the rTMS treatments, and the stimulation target was marked with a vitamin E capsule. The subjects were instructed to relax with their eyes closed and not move their heads, while remaining awake during the MRI scan. The subjects were later asked whether they had fallen asleep. Functional images were acquired using an Echo planar imaging sequence (repetition time, 2000 ms; echo time, 30 ms; flip angle, 90°; field of view, 240 × 240 mm^2^; matrix, 64 × 64; slice thickness, 5 mm; and 33 axial slices). A total of 240 volumes were collected for a total scan time of 480 s. Subsequently, 3D T1-weighted anatomical images were acquired (repetition time, 8.35 ms; echo time, 3.27 ms; flip angle, 12°; field of view, 240 × 240 mm^2^; matrix, 256 × 256; slice thickness, 1 mm; and 156 sagittal slices).

### Image pre-processing

Images were pre-processed using DPARSF v2.3 software (www.restfmri.net). The first ten functional images were excluded, and subsequent images were corrected for temporal differences by slice-timing and for head motion by alignment. The head motion parameters (three translations and three rotations) for each volume were obtained using rigid body transforms. The subject’ data were excluded from further analysis if either their translation or rotation parameters for any single volume exceeded ± 3 mm or ± 3° (e.g., movement over a voxel’s width). We also calculated the individual mean frame-wise displacement (FD) by summing the absolute values of the differentials of the six realignment parameters to express the relative transformation parameters^31^. After the skull stripping pre-procedure using the brain extraction tool in MRIcroN (www.nitrc.org/projects/mricron), individual 3D T1-weighted anatomical images were co-registered to the functional images. The co-registered 3D T1-weighted images were segmented into gray matter, white matter, and cerebrospinal fluid using SPM8 (www.fil.ion.ucl.ac.uk/spm). This segmentation employs a mixture model cluster analysis to identify voxel intensities matching particular tissue types combined with an a priori knowledge of the spatial distribution of these tissues. The segmented gray matter was then normalized to the Montreal Neurological Institute (MNI) space. These spatial transformation parameters were then applied to the functional images. The normalized fMRI data were re-sliced at a resolution of 3 × 3 × 3 mm^3^ and spatially smoothed with a 6 mm full width Gaussian kernel at half-maximum. Several sources of spurious variance (24 head motion parameters derived by Volterra expansion that included six head motion parameters, six head motion parameters one time point before, and the 12 corresponding squared items^32^, averaged signals from the cerebrospinal fluid and white matter, and the global brain signal) were eliminated by multiple linear regression^33,34^. Functional images with linear trends were removed using temporal bandpass filtering (0.01–0.08 Hz). Finally, because FC is sensitive to the confounding factor of head motion, scrubbing regression was performed for motion correction to reduce the negative influence^35^.

### Region of interest (ROI)-based FC

We examined whether FC between the stimulated left DLPFC (rTMS target) and left NAcc (DBS target) was associated with an improvement ratio in depressive and anxious symptoms. The mechanism and anti-depression effect of rTMS treatment for MDD are different between left and right DLPFC targets^8^. Considering the right and left NAcc was reported to modulate an area ipsilateral to the stimulation^36^, we had chosen the left NAcc, which is ipsilateral to left DLPFC, as a ROI to minimize the bias from the bilateral NAcc ROI. Specifically, the position of the vitamin E capsule on the scalp in the T1 image was warped into the MNI space using rigid body transformation parameters obtained via unified segmentation. Then, the center site of the vitamin E capsule on the scalp was dilated until it overlapped with the whole-brain mask. A sphere (radius = 3 mm) located at the centroid of the overlap area was used to represent the left DLPFC ROI, which directly corresponded to the stimulated target specific to individual. These steps were conducted using an in-house toolbox. We calculated the temporal correlation coefficients between the stimulated left DLPFC ROI and the left NAcc as a 3 mm radius sphere located at the MNI coordinates −9, 9, and −8^37^. The *r* values were converted using Fisher’s *r*-to-*z* transformation to make the distribution more Gaussian.

### Whole-brain FC

We further computed the whole-brain FC pattern of the left NAcc. Specifically, we calculated the temporal correlation coefficients between the left NAcc and the remaining voxels in the brain. Then, we compared the whole brain FC maps (Fisher’s *r*-to-*z* transformed maps) between early improvers and early non-improvers.

### Regional brain activity and local synchronization

We computed the regional brain activity and local synchronization of each voxel using the ALFF^25^ and ReHo^26^, respectively. The details of the ALFF and ReHo calculation can be found in the Supplementary Materials. The ALFF and ReHo maps were *Z*-standardized by subtracting the mean from the value of each voxel and then dividing by the standard deviation. We extracted the *Z*-standardized ALFF and ReHo values of the stimulated left DLPFC and left NAcc using spheres (radius = 3 mm).

### Statistical analyses

We described the relative position of the stimulated target specific to individual using the Euclidean distance between the coordinates of the stimulated target and the center of the targets across all patients. These Euclidean distances were compared between early improvers and non-improvers using the Mann–Whitney *U*-test.

We compared the age, education, age of onset, mean FD, duration of depressive episode, HAMD scores, and HAMA scores between the early improvers and non-improvers using the Mann–Whitney *U*-test. Sex and onset frequency were analyzed using the Chi-square test.

The intrinsic FC strength (Fisher’s *r*-to-*z* transformed) of the stimulated left DLPFC–NAcc within each group was compared using the Wilcoxon signed-rank test. In addition, the between group comparison of the FC strength was performed using the Mann–Whitney *U*-test. We performed correlation analyses between the FC of the stimulated left DLPFC–NAcc and either the depressive symptom (HAMD) improvement ratio or the anxious symptom (HAMA) improvement ratio using both Pearson’s and Spearman’s correlations. Then, we compared the correlation coefficients of the FC strength and HAMD (HAMA) improvements between early improvers and non-improvers using a classic interaction linear model according to previous work^38^ via SurfStat Toolbox (www.math.mcgill.ca/keith/surfstat/#ICBMagain).

Furthermore, the group-level whole brain FC patterns of the left NAcc were determined using the one-sample *t*-test for the within-group comparisons (within the gray matter mask) for each group (*p* < 0.05) with the REST software (v1.8, www.restfmri.net). A nonparametric permutation test (5000 times) was performed on the individual whole-brain FC maps between groups. The group comparisons were restricted (masked) to the voxels within the corresponding group whole-brain FC maps. The mask was created by combining the regions of significant FC patterns of the left NAcc in any group (union set), which were obtained from the one-sample t-tests results. The significance threshold was set to an AlphaSim-corrected *p* < 0.05 (height threshold *p* < 0.01; cluster extent threshold *k* > 60 voxels).

The ALFF and ReHo values of the left NAcc and the stimulated DLPFC between the early improvers and non-improvers were compared using the Mann-Whitney U-test. We performed correlation analyses between the ALFF values of the left NAcc and the stimulated DLPFC and HAMD and HAMA improvements using both Pearson’s and Spearman’s correlations. The same procedure was performed for the ReHo values.

## Results

### Patient characteristics

**Fig. 1:**
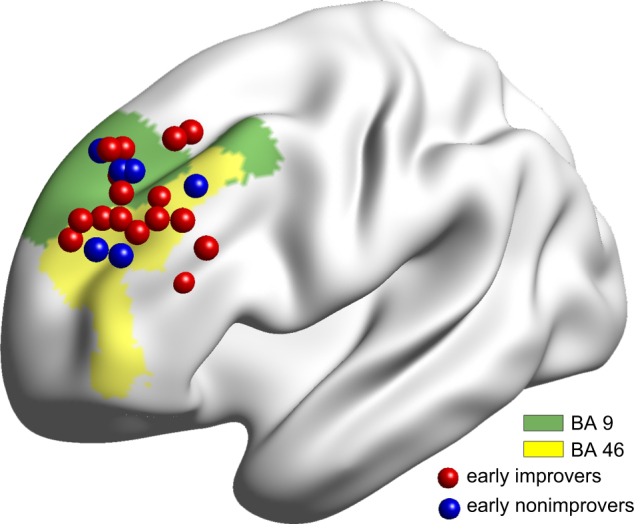
Spatial location of rTMS targeting the left DLPFC according to the ‘5 cm rule’. Most rTMS targets located in BA 46 and BA 9. The early improvers (*n* = 16) and early non-improvers (*n* = 6) are marked with red and blue, respectively

No subjects were excluded due to single volume head motion exceeding 3 mm in translation or 3° in rotation during scanning. Furthermore, the largest mean FD of all patients was less than 0.3 mm, and there was no significant difference in the mean FD between the early improvers and non-improvers (*U* = 33, *p* = 0.28). Of the 22 patients, 16 (72.73%) were early improvers, and 6 (27.27%) were non-improvers. After projecting the labeled site on the scalp to the cortex, we found that the ‘5 cm method’ primarily stimulated BA 9 or BA 46 (Fig. 1). There was no group difference in the Euclidean distance between the coordinates of the stimulated target and the center of all the targets (*U* = 45, *p* = 0.86).

There was a significant difference in age (*U* = 16.5, *p* = 0.02) between the early improvers and the non-improvers. The two subgroups did not significantly differ in education (*U* = 24.5, *p* = 0.11), FD (*U* = 33, *p* = 0.28), sex (*p* = 0.28), onset frequency (*p* = 0.20), pre-rTMS HAMA scores (*U* = 43.5, *p* = 0.76) and pre-rTMS HAMD scores (*U* = 47.5, *p* = 0.99). After the rTMS treatments, early improvers showed significantly decreased HAMA (*U* = 19.5, *p* = 0.03) and HAMD (*U* = 11, *p* = 0.004) scores compared with those of non-improvers (Table 1).

**Table 1:** Demographic and clinical characteristics of patients

Demographics	Early improvers (*n* = 16)	Early non-improvers (*n* = 6)	*P* value
Age (years)	45.00 ± 13.85	28.33 ± 10.84	0.02^a^
Sex (male/female)	6/10	3/3	0.60^b^
Education (years)	9.40 ± 2.70	11.83 ± 2.71	0.11^a^
Age of onset (years)	38.81 ± 13.98	26.17 ± 10.59	0.08^a^
first-episode/recurrence	9/7	4/2	0.66^b^
Duration of depressive episode (months)	80.69 ± 116.3	27.25 ± 21.67	0.53^a^
HAMD			
Pre-rTMS	18.50 ± 6.13	17.17 ± 1.45	0.97^a^
Post-rTMS	10.94 ± 4.00	15.83 ± 1.47	0.004^a^
HAMA			
Pre-rTMS	20.19 ± 8.55	21.17 ± 6.44	0.76^a^
Post-rTMS	10.94 ± 6.00	16.83 ± 5.00	0.03^a^

*HAMD* Hamilton Rating Scale for depression, *HAMA* Hamilton Anxiety Rating Scale, *rTMS* repetive transcranial magnetic stimulation

The values are illustrated as mean ± SD

^a^Mann Whitney test

^b^Chi-square test

### FC between DLPFC and NAcc

**Fig. 2:**
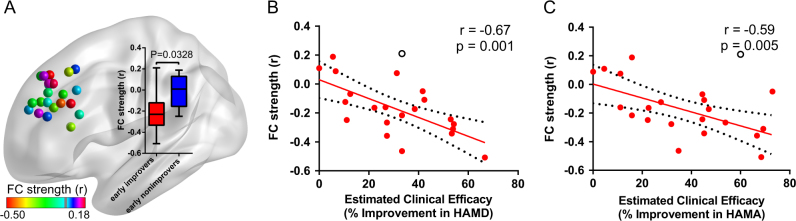
The functional connectivity between the stimulated left DLPFC and left NAcc (MNI coordinates: −9, 9, −8). The subjects are marked with different colors according to stimulated DLPFC–NAcc FC strength **a** The inset box-and-whisker plot indicates the statistical differences of FC between groups. The upper and lower whiskers denote the largest and smallest values, respectively. The early improvers exhibited increased negative FC with respect to early non-improvers (Mann–Whitney *U*-test, *U* = 19.0, *P* = 0.03). The scatter-plot indicates a significant negative correlation between the left DLPFC–NAcc FC strength and the HAMD-17 improvement ratio **b** and the HAMA-14 improvement ratio **c** Filled circles denote data points included in the correlation; open circles denote outliers. Solid lines and dashed lines represent the best-fit line and the 95% confidence interval of Pearson’s correlation coefficient, respectively

Early improvers showed increased negative FC between the stimulated left DLPFC and left NAcc (*r* = −0.21 ± 0.18, Wilcoxon signed-rank test *W* = −118, *p* = 0.001), with respect to the non-improvers (*U* = 19.0, *p* = 0.03, Fig. 2a). Furthermore, the FC between the stimulated left DLPFC and left NAcc was negatively correlated with the improvement ratios of depression (Pearson’s correlation: *r = *−0.67, *p = *0.001; Spearman’s correlation: Rho = −0.64, *p* = 0.002) and anxiety symptoms (Pearson’s correlation: *r = *–0.59, *p = *0.005; Spearman’s correlation: Rho = –0.56, *p* = 0.008) (Fig. 2b, c). When we computed the correlation in each group separately, there was no significant difference (Supplementary Fig. S2). In addition, compared to non-improvers, early improvers did not exhibit a significant correlation between FC strength and HAMD improvement (*t*_20_ _=_ 0.11, *p* = 0.91) and HAMA improvement (*t*_20_ _=_ −1.45, *p* = 0.16) using the interaction linear model (Supplementary Fig. S2).

**Fig. 3:**
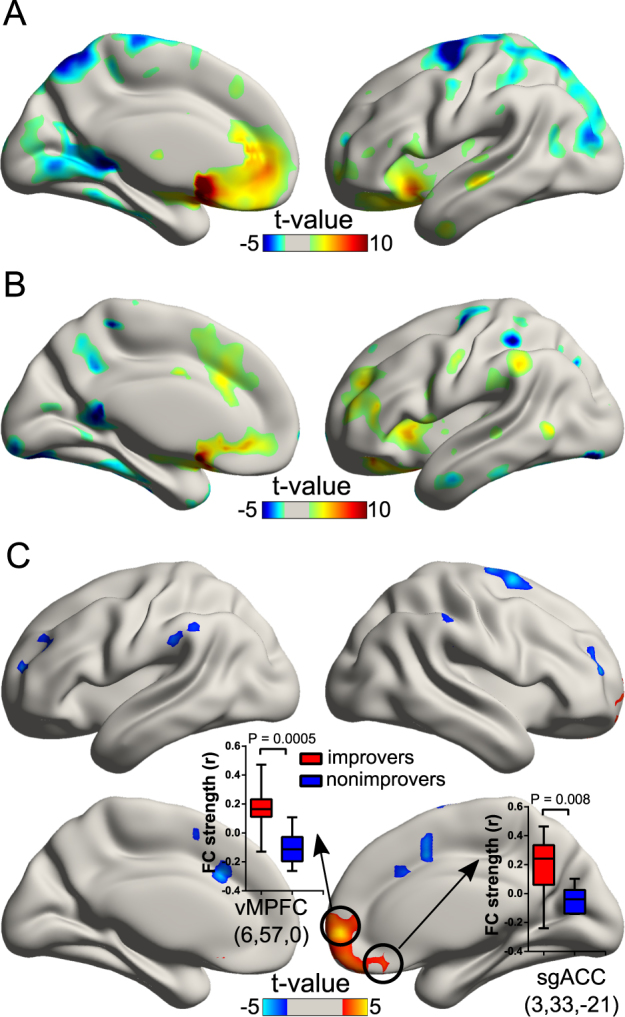
The whole brain functional connectivity patterns of the left NAcc. The whole brain FC map of the early improvers **a** and non-improvers **b**. The group differences of whole brain FC patterns of the left NAcc **c**. The inset box-and-whisker plot indicates the statistical differences of FC between groups. The upper and lower whiskers denote the largest and smallest values, respectively. The early improvers showed higher FC strength between NAcc and vmPFC (MNI coordinates: 6, 57, 0), as well as NAcc and sgACC (MNI coordinates: 3, 33, −21) than in early non-improvers. *MNI* Montreal Neurological Institute, *NAcc* nucleus accumbens, *sgACC* subgenual anterior cingulate cortex, *vmPFC* ventralmedial prefrontal cortex

Fig. 3a, b illustrates the whole-brain FC patterns of the left NAcc in each group. The left NAcc exhibited a distributed pattern of negative FC with superior parietal regions, occipital cortices and portions of the temporal cortices. The ventral lateral PFC, orbitofrontal cortex, ACC and inferior/middle temporal gyrus exhibited significantly positive FC with the left NAcc. Visual inspection shows that these patterns are similar in both groups. Statistically, the early improvers exhibited higher positive FC between the NAcc and ventralmedial PFC (vmPFC) (*U* = 5.0, *p* = 0.0005), as well as the NAcc and sgACC (*U* = 13.0, *p* = 0.008) than the non-improvers (Fig. 3c).

### Regional brain activity and local synchronization

**Fig. 4:**
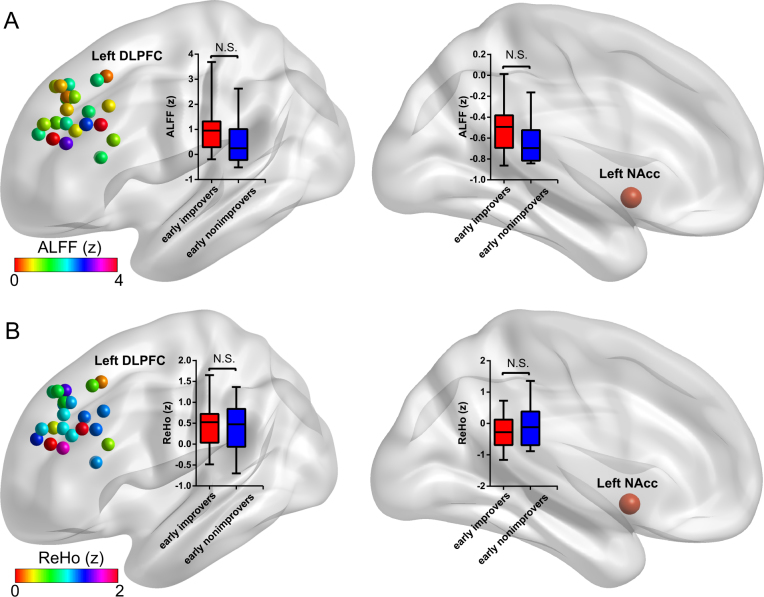
The comparisons of regional brain activity and local synchronization between early improvers and early non-improvers in the stimulated left DLPFC and the left NAcc. The regional brain activity the stimulated left DLPFC across subjects is marked with different colors according to individual ALFF value **a**. The local synchronization the stimulated left DLPFC across subjects is marked with different colors according to individual ReHo value **b**. The early improvers did not exhibit significant differences in ALFF and ReHo compared to that of the early non-improvers in the stimulated left DLPFC (left) and left NAcc (right). The inset box-and-whisker plot indicates the statistical differences of ALFF (or ReHo) between groups. The upper and lower whiskers denote the largest and smallest values, respectively. *ALFF* amplitude of low-frequency fluctuation, *DLPFC* dorsolateral prefrontal cortex, *NAcc* nucleus accumbens, *ReHo* regional homogeneity

Compared to non-improvers, early improvers did not exhibit a significant difference in intrinsic regional brain activity (ALFF) (Fig. 4a) and local synchronization (ReHo) (Fig. 4b) either in the stimulated left DLPFC or left NAcc. Furthermore, the ALFF (or ReHo) values of the stimulated left DLPFC or left NAcc did not exhibit a significant correlation with the HAMD improvement and HAMA improvement (data not shown).

### FC without global signal regression

To validate our findings, we carried out auxiliary analyses as follows. When we recomputed the ROI-based FC of the DLPFC–NAcc without global signal regression, we found that both early improvers and non-improvers showed lower FC strength between the stimulated left DLPFC and left NAcc (early improvers: *r = *−0.05 ± 0.16, non-improvers: *r = *−0.10 ± 0.11). Compared to non-improvers, early improvers did not exhibit significant differences, but did show a lower tendency (*U = *24, *p* = 0.08) (Supplementary Fig. S3A). Moreover, the stimulated left DLPFC–NAcc exhibited a significantly negative correlation with the HAMD improvement (Pearson’s correlation: *r* = −0.55, *p* = 0.01, Spearman’s correlation: Rho = −0.53, *p* = 0.01) (Supplementary Fig. S3B) and HAMA improvement (Pearson’s correlation: *r* = −0.59, *p* = 0.006, Spearman’s correlation: Rho = −0.62, *p* = 0.003) (Supplementary Fig. S3C).

### FC between DLPFC and sgACC

Furthermore, we used the sgACC (3 mm radius, MNI coordinates: −4.21, 26.2, −8.21)^16^ as the control ROI to compute the stimulated left DLPFC-sgACC FC. Compared to non-improvers, early improvers did not exhibit significant differences in the stimulated left DLPFC-sgACC FC strength (*U = *37, *p* = 0.45) (Supplementary Fig. S4A). Furthermore, the FC between the stimulated left DLPFC and sgACC did not exhibit a significant correlation with the HAMD improvement (Pearson’s correlation: *r* = −0.17, *p* = 0.48, Spearman’s correlation: Rho = −0.06, *p* = 0.81) (Supplementary Fig. S4B) and HAMA improvement (Pearson’s correlation: *r* = −0.20, *p* = 0.41, Spearman’s correlation: Rho = −0.14, *p* = 0.54) (Supplementary Fig. S4C).

## Discussion

rTMS is an effective treatment for drug-resistant MDD^2–4^ and exerts an augmented anti-depressant effect to psychotropics^24^. The goal of this study was to recognize the pretreatment brain imaging characteristics of MDD patients who would respond well to rTMS. Some previous studies have found that in major depression, brain activity in regions induced by a word generation task may predict the clinical response to rTMS^39,40^. Resting-state fMRI, which is more convenient for patients than task-related fMRI, has been widely used to predict the effects of anti-depressants, psychotherapy^41^ and rTMS^42^. Most of these studies used regional brain activity, FC or some intrinsic network as the analytic index^43^. Nevertheless, different regions and networks of interest could contribute to the varied conclusions. rTMS is a brain stimulation technology that directly affects the cortex under the coil and indirectly affects remote regions, but few studies have regarded the stimulated target as the ROI. The present study is the first to use pretreatment resting-state regional and FC features of the stimulated target to predict the anti-depression and anti-anxiety effects of rTMS. Although we used the same ‘5 cm method’ to locate the left DLPFC, the rTMS targets exhibited large variation in MNI coordinates across subjects, which further confirms the importance of focusing on the coordinates of stimulated targets instead of the coordinates described in the literature.

Two previous resting-state fMRI studies found that depressive subjects displayed lower ReHo in the left DLPFC^44,45^. However, previous task-related fMRI studies have made contradictory conclusions. Some studies showed reduced brain activation in the left DLPFC in MDD subjects relative to healthy subjects when performing an emotional matching task^46^. Other studies found higher BOLD responses in the left DLPFC when patients were exposed to implicit emotional stimuli^47^ or in a working memory task^48^. Moreover, a meta-analysis revealed that anti-depressants increased activity in the DLPFC when patients experienced both negative and positive emotions^49^. Additionally, MDD participants demonstrated reduced striatal activation compared with that of healthy controls, and the left NAcc demonstrated greater activation than the right NAcc during an affective reactivity fMRI task^50^. All of the above studies emphasize the importance of the left DLPFC and NAcc in MDD, particularly the left NAcc^51^. A previous rTMS study with only 13 subjects found that responders had greater baseline resting-state blood flow in the left DLPFC than did the non-responders, but the ROI-based analyses did not reveal any differences in the estimated stimulated area^52^. In this study^52^, the ROIs were defined by placing a 5 mm diameter sphere on published MNI coordinates of a dorsolateral prefrontal region corresponding to the EEG F3 position^52^, which does not represent the stimulated site of rTMS. Thus, we analyzed the regional cerebral activity and local synchronization of the stimulated site in the left DLPFC by labeling the site with a Vitamin E capsule, as well as the left NAcc. We did not find any significant differences in the baseline ALFF or ReHo between the early improvers and non-improvers. This result illustrates that the ALFF and ReHo in the stimulated left DLPFC and left NAcc might not be sensitive predictors of rTMS effectiveness, although this conclusion needs further confirmation.

Interestingly, the FC analyses found that early improvers showed significantly higher negative connectivity between the stimulated target and left NAcc, and higher positive connectivity of the left NAcc with ipsilateral vmPFC and sgACC than non-improvers. Previous anti-depressant studies reported consistent findings in which treatment-induced changes in the sustained engagement of the fronto-striatal circuitry track the experience of positive emotion in daily life^53^. Indeed, many fMRI studies have found abnormal connectivity in the left DLPFC of MDD patients, though notably, most of those studies focused on a particular network^41^, such as the executive control network. Several studies^50,54,55^ have revealed impaired FC between the NAcc and PFC in depression. One study found reduced fronto-striatal activity during the anticipation of gains and losses in depressed individuals^54^. Some studies used fMRI and found possible bottom-up or top-down dysfunctions of the reward system during rewarding^55^ or emotional tasks^56^. An additional study further demonstrated that disrupted topological organization within reward circuits, which are primarily located in the prefrontal-striatal regions, is significantly associated with cognitive deficits and depression severity in MDD patients^57^. However, few studies have explored the resting-state FC strength specificly between the left DLPFC and left NAcc, particularly between the stimulated target in the left DLPFC and the left NAcc. An animal study of DBS in the NAcc showed stimulation-evoked activation in the ipsilateral PFC, insula, cingulate and bilateral parahippocampal region to the NAcc^36^. Furthermore, as the stimulation voltage increased, the region of BOLD signal modulation increased in the insula, thalamus, and parahippocampal cortex and decreased in the cingulate and PFC^36^. Those researchers demonstrated that stimulation of the right and left NAcc modulated an identical area ipsilateral to the stimulation site^36^. This is why we chose the left NAcc, which is ipsilateral to the left DLPFC. Our findings supports that the FC of stimulated target with deeper brain regions, rather than regional brain activity or local synchronization of stimulated target, could predict the effects of rTMS. Left NAcc could be one of the most important deeper brain regions. Nevertheless, those responders had greater positive FC of left NAcc with vmPFC and sgACC than non-responders, rather than left DLPFC. It is possible that left DLPFC is not the target with best efficacy, but the most practical target in consideration of its position available and efficacy.

In general, the fact that negative connectivity predicted a good response to treatment suggests that positive connectivity is adaptive and might be induced by TMS. This finding counters the dominant model that negative correlations are good because they indicate DLPFC suppression of subcortical structures. Our findings support a model of integration of information between the DLPFC and mesolimbic areas as adaptive for emotional experiences^58^ and, thereby, depression^59^, although some would argue is associated with positive FC^60^.

Fox et al. provided evidence that the anti-depression efficacy of rTMS depends on the left DLPFC-sgACC FC strength^42^. We calculated the ROI-based FC between the sgACC and stimulated regions in the left DLPFC, but our findings showed there was no obvious association between the FC strength and improved HAMD and HAMA scores, which does not fit Fox’s hypothesis. Considering the small sample size in this study, we are unable to draw the definitive conclusion that the NAcc is more important than the sgACC for predicting the rTMS effects in MDD. It is possible that this small sample just represent those patients with anxiety features other than all depression patients, particularly given that most of our depression patients had relatively evident anxiety symptoms. We prudently speculated that the baseline FC of the stimulated left DLPFC–NAcc might be a more sensitive predictor than that of the left DLPFC-sgACC in depression patients with anxiety features. however, this hypothesis still needs to be validated.

There are some limitations to the current study. First, our findings are based on a small cohort of patients. Second, we regarded the site labeled by the Vitamin E capsule as the actual stimulated target of the high frequency rTMS, which reflects the stimulated cortex to the greatest extent. Actually, there may have been errors induced by the placement angle of the coil, the distance between the scalp and the cortex, and movement of the Vitamin E-labeled hat during the fMRI scan. Third, this study lacks a normal control group and post-treatment fMRI information, which may help to further validate our conclusions. These issues prevent the applicability of these findings to clinical interventions. Nonetheless, this work strongly suggests that an individual resting-state FC analysis is a feasible method for optimizing the rTMS target in MDD patients.

Using high-frequency rTMS to the left DLPFC in medication-naïve MDD patients wearing a hat labeled with a Vitamin E capsule, we demonstrated for the first time that the anti-depression and anti-anxiety effects of rTMS were correlated with the strength of the resting-state FC between the stimulated targets and the left NAcc at baseline, rather than the regional brain activity or local synchronization in these two brain regions. These findings suggested intrinsic FC might help to optimize individual rTMS targets. In future research, we advise using multiple fMRI methods to detect regional activity combined with structural connectivity to verify these conclusions, as well as an exploration of the post-treatment influence of rTMS on brain images.

## Electronic supplementary material

Supplemental Material
