# Supplementary material for: Stimulated left DLPFC-nucleus accumbens functional connectivity predicts the anti-depression and anti-anxiety effects of rTMS for depression
Source: Transl Psychiatry. 2018 Mar 9;7:3. doi: 10.1038/s41398-017-0005-6 (PMC5843586; doi:10.1038/s41398-017-0005-6)
Supplement: Supplementary file 1 — Supplemental Material [file 41398_2017_5_MOESM1_ESM.pdf]

Supplementary material for

"Stimulated left DLPFC-nucleus accumbens functional connectivity predicts the anti-depression and anti-anxiety effects of rTMS for depression "

Running title: Brain connectivity predicts the effects of rTMS for depression

Author names and affiliations:

Lian Du[1]; Huan Liu[1]; Wanyi Du[1]; Fenglei Chao[2]; Lei Zhang[2]; Kejian Wang[3]; Chunxia Huang[4]; Yuan Gao[5]; Yong Tang[2]*

[1]Department of Psychiatry, the First Affiliated Hospital of Chongqing Medical University, Chongqing 400016, P.R. China

[2]Department of Histology and Embryology, Chongqing Medical University, Chongqing 400016, P.R. China

[3]Department of Anatomy, Chongqing Medical University, Chongqing 400016, P.R. China

[4]Department of Physiology, Chongqing Medical University, Chongqing 400016, P. R. China

[5]Department of Geriatrics, The First Affiliated Hospital of Chongqing Medical University, Chongqing 400016, P. R. China

Conflicts of Interest and Source of Funding:

No conflicts of interest to declare.

*Corresponding author:

Dr. Yong Tang, Department of Histology and Embryology, Faculty of Basic Medical Sciences, Chongqing Medical University.

Phone: +86-13883933846. Fax: +86-23-68485868. E-mail: ytang062@163.com

ALFF

Image preprocessing

Images were pre-processed using DPARSF v2.3 software (www.restfmri.net). The first ten functional images were excluded, and subsequent images were corrected for slice-timing and alignment. We corrected the remaining images for head motion. No subjects were excluded due to head motion exceeding 3mm in translation or 3° in rotation during scanning. We also calculated individual mean frame-wise displacement (FD) by translation and rotation parameters of head motion based on the formula from a previous study[1]. No significant differences were found between groups(P = 0.28). Individual 3D T1-weighted anatomical images were co-registered to the functional images. The 3D T1-weighted images were segmented into gray matter, white matter, and cerebrospinal fluid and then normalized to Montreal Neurological Institute space. These spatial transformation parameters were then applied to the functional images. Normalized data were re-sliced at a resolution of $3 \times 3 \times 3$ mm^3 and spatially smoothed with a 6 mm full width Gaussian kernel at half-maximum. Several sources of spurious variance (24 head motion parameters, averaged signals from the cerebrospinal fluid and white matter, the global brain signal, and head motion scrubbing regressors) were eliminated by multiple linear regression[2, 3]. Functional images with linear trends were removed using temporal band-pass filtering (0.01–0.08 Hz).

Regional brain activity analysis

We used ALFF to analyze the regional brain activity at each voxel[4]. The time series for each voxel were transformed to the frequency domain using Fast Fourier Transform, and the power spectrum was then obtained. Then the square root was calculated at each frequency of the power spectrum, and the averaged square root was obtained across 0.01-0.08 Hz at each voxel. The averaged square root was considered as the ALFF. Finally, we extracted the ALFF values in NAcc and DLPFC across subjects.

The ALFF values in left NAcc and the stimulated targets DLPFC between early-improvers and early non-improvers were compared using Mann-Whitney U-tests.

ReHo

Image preprocessing

Images were pre-processed using DPARSF v2.3 software (www.restfmri.net). The first ten functional images were excluded, and subsequent images were corrected for slice-timing and alignment. We corrected the remaining images for head motion. No subjects were excluded due to head motion exceeding 3mm in translation or 3° in rotation during scanning. We also calculated individual mean frame-wise displacement (FD) by translation and rotation parameters of head motion based on the formula from a previous study[1]. No significant differences were found between groups (P = 0.28). Individual 3D T1-weighted anatomical images were co-registered to the functional images. The 3D T1-weighted images were segmented into gray matter, white matter, and cerebrospinal fluid and then normalized to Montreal Neurological Institute space. These spatial transformation parameters were then applied to the functional images. Normalized data were re-sliced at a resolution of 3 × 3 × 3 mm^3. Several sources of spurious variance (24 head motion parameters, averaged signals from the cerebrospinal fluid and white matter, the global brain signal, and head motion scrubbing regressors) were eliminated by multiple linear regression[2, 3]. Functional images with linear trends were removed using temporal band-pass filtering (0.01–0.08 Hz).

Local synchronization analysis

We also used ReHo to analyze the local synchronization at each voxel. We used the DPARSF v2.3 software (www.restfmri.net) to calculate the time series of every voxel and its nearest voxels for regional homogeneity to obtain the individual ReHo map. Finally, we extracted the ReHo values in NAcc and DLPFC across subjects.

Supplementary Figure S1

Figure S1. A consort flowchart about exclusion of participants and the reasons.

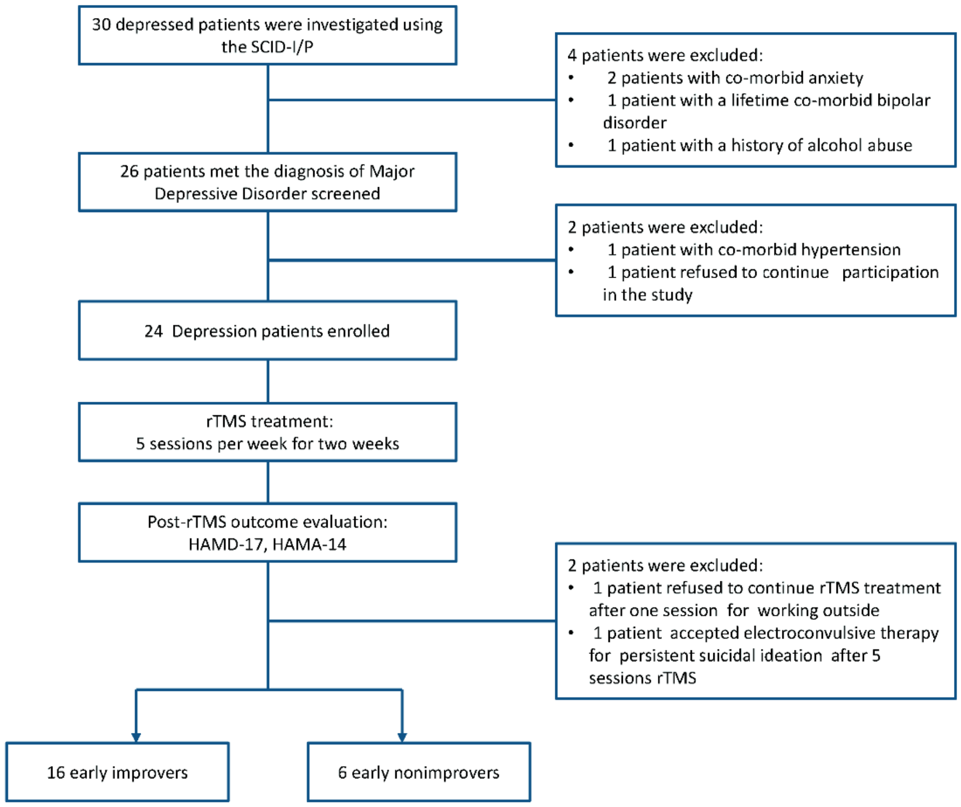

Supplementary Figure S2

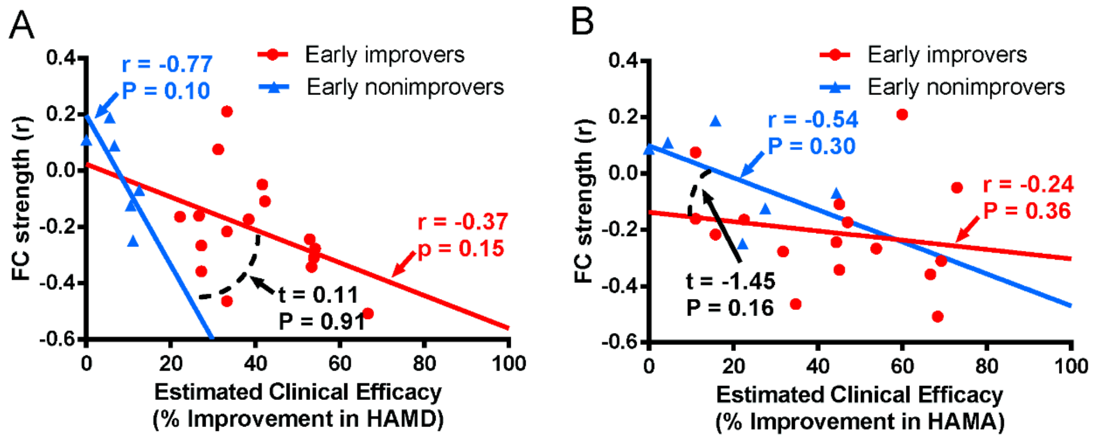

Figure S2. The comparison of Pearson's correlation coefficients between early improvers and early non-improvers. Early improvers did not exhibit correlation differences between FC strength and HAMD (t_{20} = 0.11, p = 0.91) and HAMA (t_{20} = -1.45, p = 0.16) improvement compared to early non-improvers. The red and blue scatter-plot represented early improvers and early non-improvers. Abbreviations: FC, functional connectivity; HAMD, 17-item Hamilton Depression Scale; HAMA, 14-item Hamilton Anxiety Scale.

Supplementary Figure S3

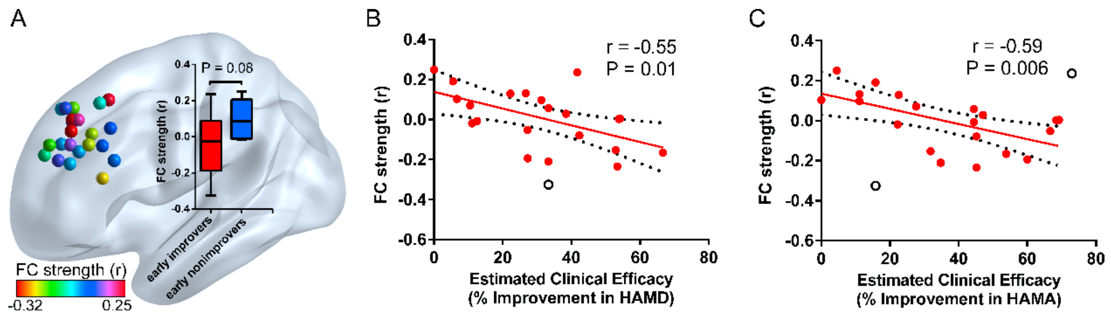

Figure S3. The functional connectivity between the stimulated left DLPFC and left NAcc (MNI coordinates: -9,9,-8) without global signal regression. The subjects are marked with different colors. The early improvers exhibited a trend higher negative correlation than the early non-improvers (P = 0.08) (A). The scatter-plot indicates a significant negative correlation between the left DLPFC-NAcc FC strength and the HAMD-17 improvement ratio (B) and a significant negative correlation between the left DLPFC-NAcc connectivity FC strength and the HAMA-14 improvement ratio (C). Filled circles denote data points included in the correlation; open circles denote outliers. Solid lines and dashed lines represent the best-fit line and the 95% confidence interval of Pearson's correlation coefficient, respectively.

Supplementary Figure S4

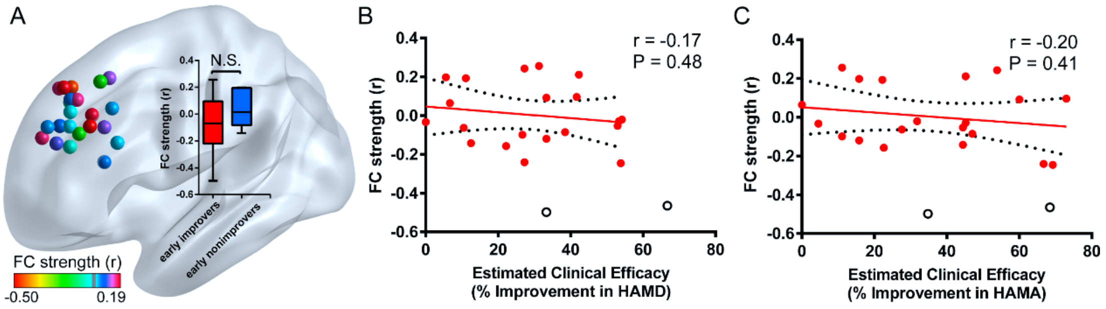

Figure S4. The functional connectivity between the stimulated left DLPFC and left sgACC (MNI coordinates: -4.21, 26.2, -8.21). The subjects are marked with different colors. The early improvers did not exhibit a significantly higher negative correlation than the early non-improvers (A). These are no significant correlation between the left DLPFC-sgACC FC strength and the HAMD-17 improvement ratio (B) as well as between the left DLPFC-sgACC FC strength and the HAMA-14 improvement ratio (C). Filled circles denote data points included in the correlation; open circles denote outliers. Solid lines and dashed lines represent the best-fit line and the 95% confidence interval of Pearson's correlation coefficient, respectively.

References

1 Power JD, Barnes KA, Snyder AZ, Schlaggar BL, Petersen SE. Spurious but systematic correlations in functional connectivity MRI networks arise from subject motion. *Neuroimage* 2012; **59**: 2142-2154.

2 Ji GJ, Yu Y, Miao HH, Wang ZJ, Tang YL, Liao W. Decreased network efficiency in benign epilepsy with centrotemporal spikes. *Radiology* 2017; **283**: 186-194.

3 Zhu Y, Yu Y, Shinkareva SV, Ji GJ, Wang J, Wang ZJ *et al.* Intrinsic brain activity as a diagnostic biomarker in children with benign epilepsy with centrotemporal spikes. *Hum Brain Mapp* 2015; **36**: 3878-3889.

4 Zang YF, He Y, Zhu CZ, Cao QJ, Sui MQ, Liang M *et al.* Altered baseline brain activity in children with ADHD revealed by resting-state functional MRI. *Brain Dev* 2007; **29**: 83-91.
